# Egocentric-View Fingertip Detection for Air Writing Based on Convolutional Neural Networks †

**DOI:** 10.3390/s21134382

**Published:** 2021-06-26

**Authors:** Yung-Han Chen, Chi-Hsuan Huang, Sin-Wun Syu, Tien-Ying Kuo, Po-Chyi Su

**Affiliations:** 1 Department of Computer Science and Information Engineering, National Central University, Taoyuan City 32001, Taiwan; 106522108@cc.ncu.edu.tw (Y.-H.C.); 108522044@cc.ncu.edu.tw (C.-H.H.); 108522096@cc.ncu.edu.tw (S.-W.S.); 2 Department of Electrical Engineering, National Taipei University of Technology, Taipei 10608, Taiwan; tykuo@ntut.edu.tw

**Keywords:** air-writing, fingertip detection, region-based convolutional neural network, smart glasses

## Abstract

This research investigated real-time fingertip detection in frames captured from the increasingly popular wearable device, smart glasses. The egocentric-view fingertip detection and character recognition can be used to create a novel way of inputting texts. We first employed Unity3D to build a synthetic dataset with pointing gestures from the first-person perspective. The obvious benefits of using synthetic data are that they eliminate the need for time-consuming and error-prone manual labeling and they provide a large and high-quality dataset for a wide range of purposes. Following that, a modified Mask Regional Convolutional Neural Network (Mask R-CNN) is proposed, consisting of a region-based CNN for finger detection and a three-layer CNN for fingertip location. The process can be completed in 25 ms per frame for 640×480 RGB images, with an average error of 8.3 pixels. The speed is high enough to enable real-time “air-writing”, where users are able to write characters in the air to input texts or commands while wearing smart glasses. The characters can be recognized by a ResNet-based CNN from the fingertip trajectories. Experimental results demonstrate the feasibility of this novel methodology.

## 1. Introduction

Wearable devices have become increasingly popular nowadays. These portable yet powerful gadgets may significantly benefit humans in the near future due to their tight connection with users. Several interesting scenarios can thus be realized, especially when wearable devices are equipped with a variety of sensors or cameras to acquire a large amount of information around users. One of the highly regarded wearable devices is smart glasses, with which egocentric or first-person-perspective views can be collected. Advanced image and video processing methods can be utilized to deal with large volumes of imagery data and facilitate real-time applications. Nevertheless, unlike a keyboard or mouse for a computer or a touch screen on a smartphone, lacking an ideal human–device interface in smart glasses could be a serious concern that limits their use. Although speech commands could be used to control smart glasses, interference from environmental noises may pose challenges to accurate command recognition. Besides, users may be reluctant to talk to machines for a variety of reasons. In the absence of a suitable way of inputting texts or commands, smart glasses are often regarded as only a type of devices for collecting visual information from users’ surrounding areas.

To make the human–device interface of smart glasses more user-friendly, hand gestures were considered in this research to provide necessary commands to smart glasses by pointing or sliding subjects with fingers and viewed from a user’s perspective. To be more specific, we aimed at allowing users to write in the air by their index fingers as the text input since such “air-writing” is quite a natural way for users to conveniently apply and can be read by users themselves. If the fingertip can be detected correctly, the writing trajectories will be formed and further recognition can be applied to correctly determine the written characters. It should be noted that many smart glasses are equipped only with RGB cameras, rather than depth cameras, due to potential cost and weight concerns. However, without the depth information, it is quite difficult to distinguish the foreground (the finger in our scenario) from the background in the smart glasses-captured scenes. Therefore, the major challenge here is to efficiently detect the fingertip in RGB images while overcoming the interference from the complex background, various illuminations, the motion-blurriness caused by fast-moving fingers or cameras, etc.

**Figure 1:**
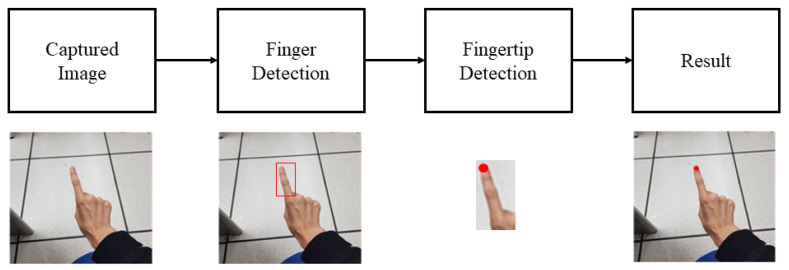
The steps of locating the fingertip in the proposed scheme.

Deep-learning approaches are adopted to achieve the above-mentioned objectives. To effectively detect the small object, i.e., fingertip, we propose a modified Mask R-CNN [1] for locating the fingertip with high accuracy in real-time. Figure 1 illustrates the proposed procedures. To begin, the finger region is identified by a bounding box detected via a region-based CNN to filter out the majority of the irrelevant background interference. Then, a second-level CNN is formed to generate a mask for determining the fingertip coordinate. The detected writing trajectories can then be used to recognize written characters.

The paper is organized as follows. Section 2 reviews the related work of hand or finger detection. Section 3 details our CNN-based fingertip detection architecture and the generation of the synthetic dataset. Section 4 describes the integration of fingertip detection with smart glasses. A character recognition model is also presented as an example to demonstrate the feasibility of the proposed scheme. Section 5 shows the experimental results. Finally, the conclusion and future work are discussed in Section 6.

## 2. Related Work of Hand Detection

Traditional methods for locating hands or fingers in images rely on manually designed feature extraction from pixel values. Skin colors, hand shapes, etc. are employed to detect the appearance of targeted objects. Since the diversity of skin colors and changes in illumination can affect the detection accuracy, Girondel et al. [2] found that Cb and Cr color channels are more suitable for the skin detection task. Sigal et al. [3] proposed the Gaussian mixture model that performed quite well under varying illumination conditions. However, the methods based on skin colors may not work if the background also has similar colors. M. de La Gorce et al. [4] pre-processed incoming frames to convert images into 3D models for hand detection and positioning. Its applications are limited due to the high computational load. Certain methods resorted to the additional depth information in frames. P. Krejov et al. [5] employed a depth camera to help remove backgrounds and then used the Dijkstra algorithm to determine possible fingertip positions. The Kalman filter was adopted to locate more accurate fingertip locations. H. Liang et al. [6] also used depth information to obtain the rough areas of targeted hands and utilized the distance transform to calculate palm contour. Three depth-based features were used to locate the coordinates of fingertips. Chen et al. [7] located and tracked the center of hands using a region-growing technique.

Deep-learning-based approaches have become increasingly popular in recent years. Despite the fact that many deep neural networks for object detection have been developed, detecting tiny objects remains challenging. In order to reduce the interference of complex backgrounds on small objects, studies have been conducted to take images and their depth information as input to train deep neural networks. For example, J. S. Supancic et al. [8] proposed using a depth camera to estimate hand pose. J. Tompson et al. [9] achieved real-time puppeteering using hand detection. L. Baraldi et al. [10] presented a method for monocular hand gesture recognition in ego-vision scenarios that can work in nearly real time on wearable devices. Wetzler et al. [11] used a CNN-based fingertip detection model with a Kinect camera. A global orientation regression approach was proposed and depth images were used to predict the position of fingertips. We can see from these examples that depth information helps to remove backgrounds while retaining meaningful foregrounds. However, the requirement of depth cameras is undoubtedly a burden, especially for current smart mobile devices that demand low cost and lightweight. In addition, some depth cameras acquire the depth information by calculating the distance between the object and the lens through estimating the return time of infrared. When such devices are operated outdoors, the natural light may also contain infrared and affect their functions.

The research on deep-learning-based hand or finger detection based on RGB images is gaining more attention. S. Bambach et al. [12] used a region-based CNN model to detect hands. It appears that the repeated computation of redundant overlapping proposals is computationally expensive. C. Xu et al. [13] proposed another CNN-based model and used a Generative Adversarial Network (GAN) to produce more realistic hand appearances to improve the detection performance. X. Liu et al. [14] used a two-stage CNN model to detect index fingertips and joint coordinates. The first-layer CNN model is responsible for detecting the bounding box of the hand, while the second layer of the CNN regression model calculates index fingertips and joint coordinates. Y. Huang et al. [15] designed a two-stage model, with the first stage using Faster R-CNN to detect the bounding box of hands in images and the second stage trying to locate index fingertips and joint coordinates. Mukherjee et al. [16] also used Faster R-CNN to select hand areas with a bounding box. An additional step was taken to find fingertip location using traditional image processing methods. M. M. Alam et al. [17] identified finger classes and positions using a single neural network. The model used ensemble averaging to obtain the final positions after regressing the positions of fingertips from a fully convolutional network.

It should be noted that, in the absence of depth information, the detection of small objects in images will be influenced by several environmental factors easily. Therefore, many models tend to be two-stage approaches, with the first step roughly finding hand areas in images, eliminating most of the background noises and the second step using another CNN model to further detect and locate fingertips. Thus, the benefit is similar to the function of depth information in reducing the negative effects of backgrounds.

## 3. The Proposed Fingertip Detection Scheme

**Figure 2:**
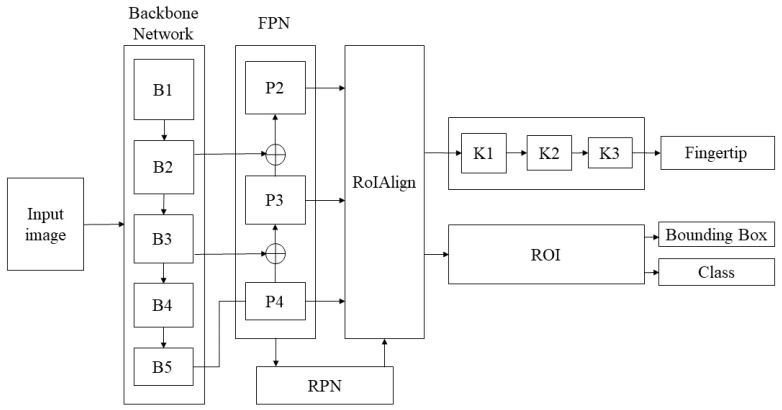
Proposed structure of fingertip detection [20].

The proposed fingertip detection method adopts a two-stage methodology as the reasons mentioned above. Figure 2 shows the adopted deep learning structure, which is divided into four parts, including the backbone network, the FPN (Feature Pyramid Network) [18], the RPN (Region Proposal Network) [19], and the three-layer CNN branch network to detect the fingertip.

### 3.1. Design of Backbone Network

**Figure 3:**
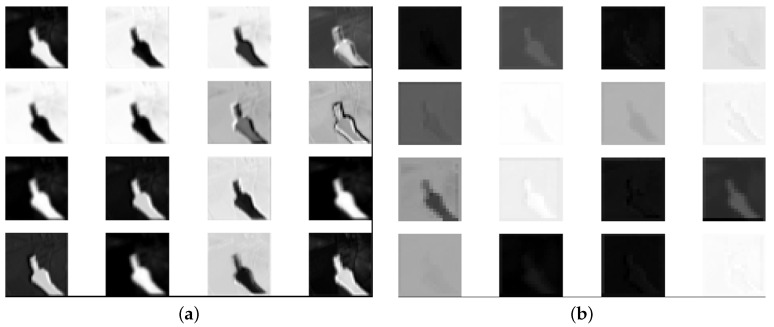
Feature maps from different bottleneck layers: (**a**) bottleneck layer No. 9; and (**b**) bottleneck layer No. 12.

The purpose of the backbone network is to extract the feature maps from input images using convolution operations. Current mainstream architectures, such as ResNet [21], DenseNet [22], and Inception networks [23], tend to adopt “deeper” designs. Such designs, however, indicate that more parameters and computations are required. To build a lightweight backbone network, we adopted the depth-wise separable convolution because it effectively reduces the computational load while sacrificing only a small amount of accuracy. To address the degradation issue, the bottleneck design in MobileNetV2 [24] is adopted. The basic idea is to expand the features first and then compress them at the end of the bottleneck block. We also observed that trimming bottleneck layers further improves the processing speed while maintaining the detection accuracy, as deeper layers may only extract redundant features in our considered cases. Figure 3 shows the feature maps from different bottleneck layers when the scheme is used to detect a hand for better illustration. Figure 3a demonstrates a clear hand in a relatively shallow layer, while Figure 3b shows less obvious features in a deeper layer. Therefore, fewer layers are used in the proposed scheme to reduce the number of trained parameters and detection time as well.

Our backbone network was set to have 9+1 layers after experiments. The design details are shown in Table 1, in which *t*, *c*, *n*, and *s* indicate the expansion factor, the number of output channels, the repeating number, and the stride, respectively. The first layer is a 1×1 convolution layer that adjusts the input size to allow the network to adapt to multi-scale inputs. The following nine layers are composed of five-dimensional bottleneck blocks.

### 3.2. Design of Object Detection Network

Feature Pyramid Networks (FPN) were employed to create multi-scale feature maps to deal with the variation of object sizes. As is known, the layers closer to the input can extract low-level features such as edges and location information, whereas the layers closer to the output tend to extract high-level features with more semantic information. It should be noted that smaller objects are more likely to vanish in deeper layers. FPN improve the accuracy of detecting small objects and may help in achieving a balance between processing speed and accuracy. The proposed scheme involves up-sampling the feature map B5 to higher resolution and then merging it with the low-level feature maps B2 and B3 based on the five-dimensional bottleneck blocks. Our FPN design outputs the feature maps with three scales, which are combined with features from various layers.

**Figure 4:**
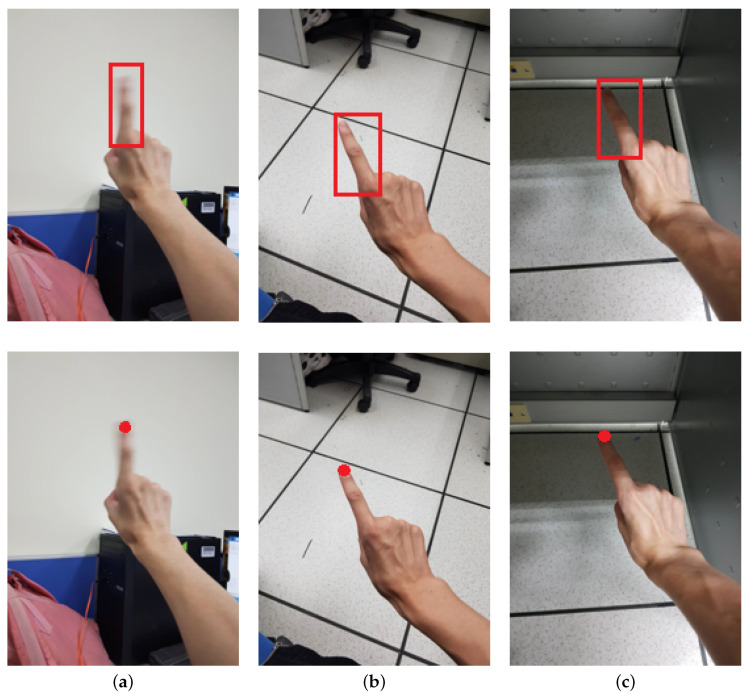
The results of finger and fingertip detection in images of: (**a**) blurred finger; (**b**) lighter scene; and (**c**) darker scene.

Region Proposal Networks (RPN) output a set of finger proposals with sliding anchors. This is followed by the region of interest align (RoIAlign) [1] to convert valid regions into fixed-size feature maps without quantization and preserve exact spatial location. This strategy can speed up the detection and maintain the localization accuracy, as illustrated in Figure 4.

### 3.3. CNN-Based Fingertip Detection

**Figure 5:**
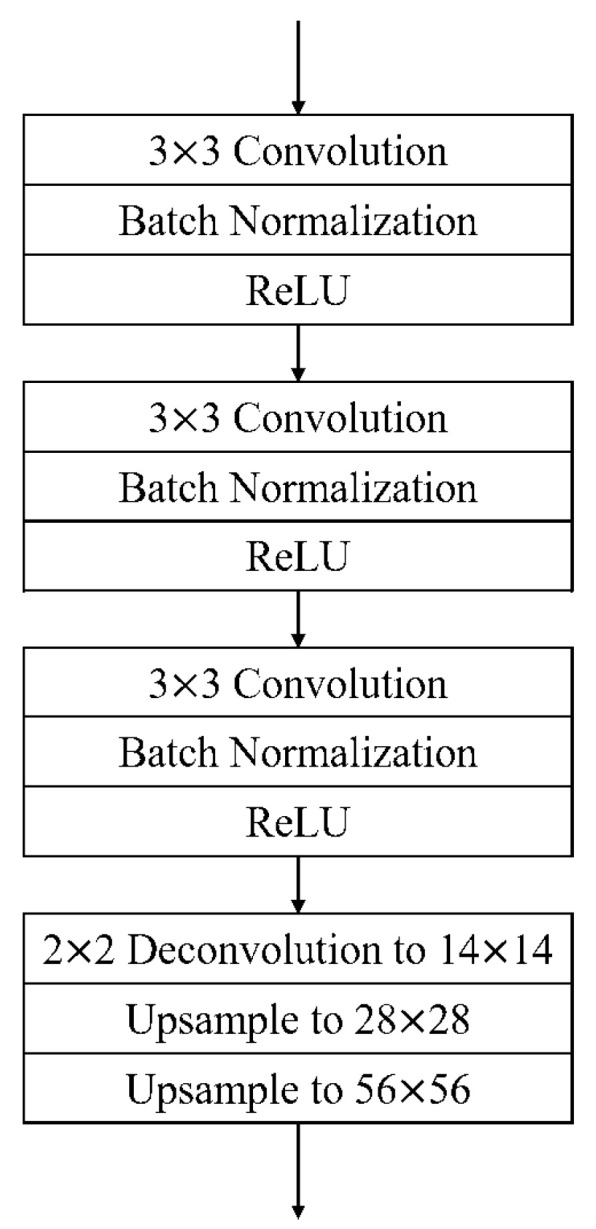
Design of the three-layer CNN.

The objective of the second-level network is to determine the accurate location of fingertips. We added a branch network at the end of the RoIAlign layer. Figure 5 shows the design of our three-layer CNN. Each feature map will go through a three-layer CNN and a 56×56 mask will be generated to identify the exact fingertip location based on the finger region.

### 3.4. Synthetic Training Dataset

The quality of training dataset has a significant impact on neural network performance. The size of datasets, the diversity of content, and the human efforts devoted to constructing a dataset, are all important factors. Creating a large training dataset can be a tedious and time-consuming task. Moreover, manual labeling errors are inevitable and may affect the results considerably.

**Figure 6:**
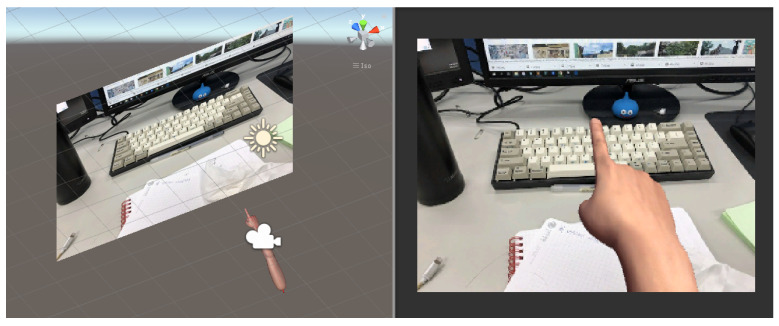
Creation of the egocentric fingertip dataset using Unity3D.

Stephan R. Richter, Vibhav Vineet, Stefan Roth, & Vladlen Koltun. [25] proposed to use modern computer games to rapidly create pixel-accurate semantic label maps for training images. Inspired by this work, we decided to create our synthetic training dataset using Unity3D. The main idea is to synthesize the 3D hand model into real-world scenes. The major advantage here is that we can use the predefined joint coordinates from the 3D hand model to generate accurate annotations, avoiding poor labeling by human annotators. Figure 6 depicts an example of a synthesized hand superimposed on an image. Since the objective of this research is to enable air-writing for smart glasses, the first-person-perspective view is considered, and it usually shows one hand with varying backgrounds. Using Unity3D to create a large volume of labeled fingertips in various images is thus a good match for the proposed scheme.

The downside of using synthetic images is that the neural network is more likely to overfit such training images and may perform poorly on real-world images. There are two strategies to solve this issue. The first method is to generate the synthetic images that are as close to real-world images as possible. In other words, the lightness, shadows, and finger positions should all be reasonable. The other different method is to generate synthetic images without considering the reasonableness and to try to increase the diversity of data as much as possible. Our experiments indicated that using diverse images has a greater influence on training the network and results in better performance than using reasonable images. Increasing diversity can effectively prevent the network from overfitting the synthetic images as well.

**Figure 7:**
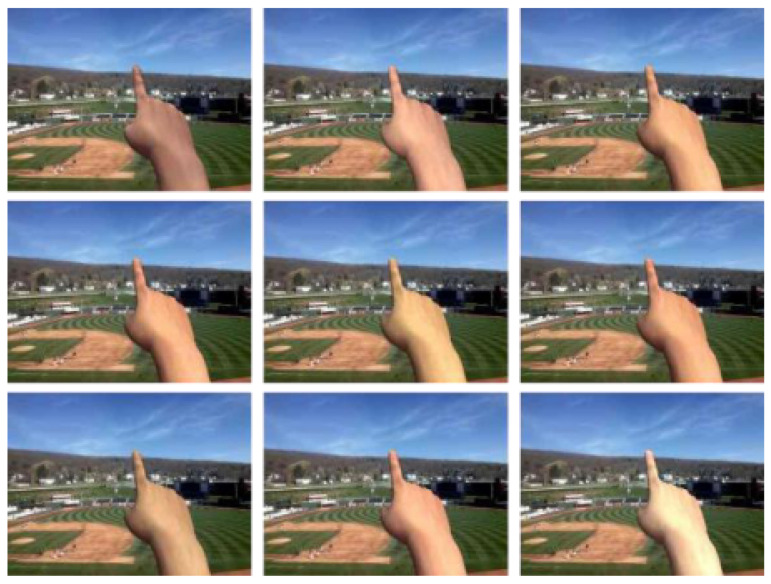
Employing different skin colors and textures to increase diversity of synthetic hands.

We changed the background brightness of each training image by applying random grey masks. We also randomized the light positions and directions to create different shadows. In addition, 13 skin colors and 4 skin textures were used to increase the diversity of synthetic hands, as shown in Figure 7. Furthermore, we randomly changed the hand model in 3D positions and directions. Motion blurriness was also added to the finger to simulate the cases of the fast-moving hand. Finally, we stored the images using lossy JPEG compression with the quality factor 50 to deal with potentially low-quality input.

**Figure 8:**
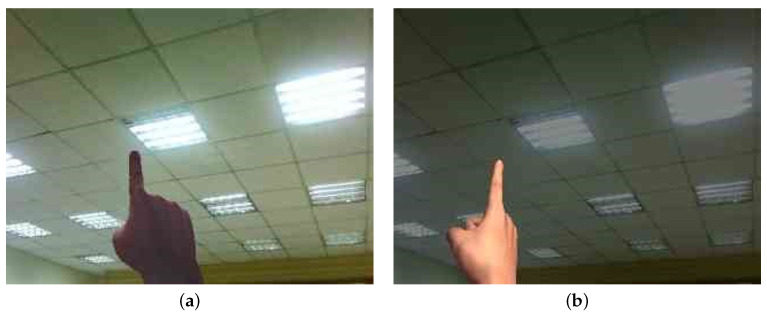
Challenging cases of fingertip detection in: (**a**) a very light environment; and (**b**) a very dark environment.

During the experiments, we found that the detection network may fail in some extreme environments, such as the cases shown in Figure 8. In Figure 8a, the smart glasses user operates hand writing towards lights. The hand becomes very dark and the detection of the fingertip may fail if the training dataset does not include such cases. On the other hand, Figure 8b shows that the user operates in a dark environment and the color of the skin may look different. To increase the robustness of the detection scheme, we generated training images to simulate such environments. This appeared to be an advantage of using synthetic images as we can create the corresponding cases according to different difficult scenes. This strategy improves the adaptability of the proposed network model to various situations.

**Figure 9:**
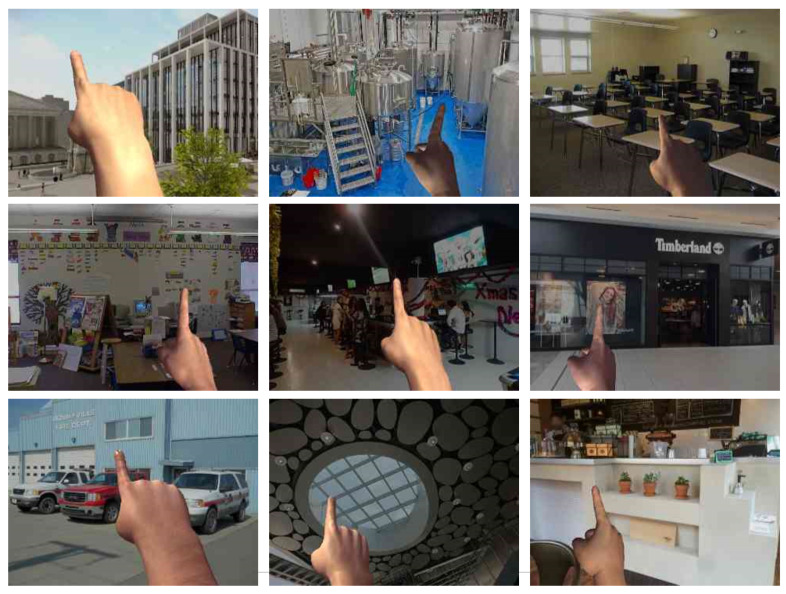
Image samples from our synthetic dataset.

Our dataset contains 311,972 images for training and 188,732 images for validation with a resolution of 640×480 pixels. All images have fingertip annotations with high accuracy. Figure 9 shows some samples from our dataset. The background images are from ImageNet [26].

## 4. System Integration

### 4.1. Integration of Fingertip Detection in Server with Smart Glasses

After the fingertip can be traced successfully, air-writing for smart glasses can be implemented. Building a neural network for fingertip detection directly on smart glasses is currently not feasible due to hardware constraints. There are thus two options. One option is to detect the fingertip in a smart phone that is connected wirelessly to the smart glasses. The other option is to send the captured scenes to the a backend server for the fingertip detection. We adopted the latter option because the former may still raise concerns about the power consumption of the smart phone. To reduce the computational burden of smart glasses, each frame is streamed using Motion JPEG. Our server platform is equipped with a single GTX 1080 Ti GPU (manufactured by NVIDIA Corp., Santa Clara, CA, USA). The frame is sent to the server by the smart glasses via wireless networks for subsequent detection and recognition. After the frame is acquired by the server, the model will locate the fingertip in real time and store the current coordinate of fingertips into an array. When the model does not detect a fingertip in a few frames, the process of storing the coordinates is halted. If the user finished writing and would like to see the results, what he or she needs to do is to move the fingertip away from the camera. The points on the writing trajectories are represented by the stored coordinates, and the character can be formed by connecting these points.

**Figure 10:**
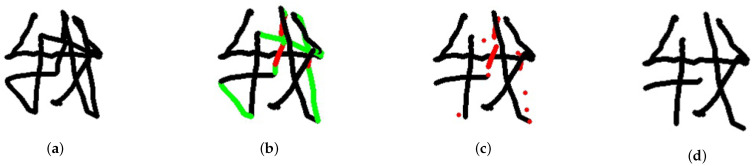
Steps involved in removing redundant connections from Chinese characters: (**a**) all trajectories appear in a single curve or stroke; (**b**) all redundant connections are found (the green segments represent the connections from the bottom right to the top left, while the red segments represent the connections from the bottom left to the top right with a steep slope); (**c**) all of the green segments are removed; and (**d**) the final results after the red segments are removed.

In this research, Chinese characters are considered the input to the smart glasses as an example, given the observation that air-writing seems a more natural way for inputting Chinese characters. Recognizing English letters from air-writing is certainly feasible, but one may argue that selecting English letters from a visual keyboard may be preferred. It is worth noting that air-writing cannot display the action of lifting pens. In other words, all of the trajectories are drawn with a single curve or stroke. Some redundant or unnecessary connections may thus appear in a character, which could cause recognition errors. Therefore, we tried to eliminate the redundant or unnecessary connections in written characters according to the following three principles. First, we removed the extra points introduced by the stopped finger during the writing process. Second, we eliminated the connections from the bottom-right to the top-left, which are not common when writing Chinese characters. Third, we cleared the connections from the bottom-left to the top-right with a steep slope, for the same reason as in the second principle. Figure 10 illustrates the detailed steps of removing redundant connections from the Chinese character, “I”.

**Figure 11:**
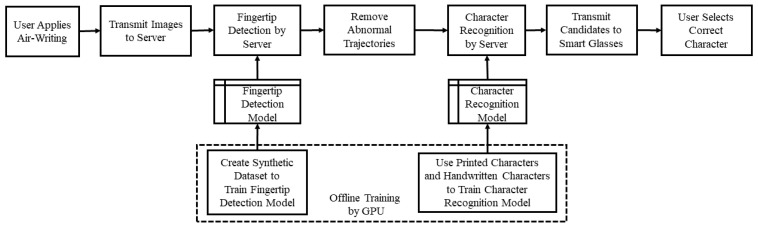
Diagram of proposed system architecture.

Finally, we sent the processed fingertip trajectories to the traditional Chinese character recognition scheme, which can return the recognized results of the top ten candidates back to the smart glasses. The candidate characters are displayed on the screen of the smart glasses for the user to select. After choosing one character, the user can move on to write the next character. If the correct character does not appear in the candidate list, the user can simply rewrite it. Figure 11 shows our overall system flow chart. The user applies air-writing and the scene is captured by the smart glasses, which transmit the images to the server. The server uses the fingertip detection model, trained by our synthetic dataset, to form the writing trajectories. After removing some redundant connections, the server recognized the character using another classification model, which is described below. The candidates are sent to the user and shown on the screen of smart glasses. The user can select a correct character (usually the first candidate) by using the button operated by the other hand, as we believe that this could be the most convenient way for smart glasses users.

### 4.2. Chinese Character Recognition Scheme

After successfully detecting the position of the fingertip, the writing trajectories through air-writing are available. One possible way is to transmit the trajectories to character recognition services such as Google Cloud Vision API. In order to reduce the dependence on using third-party software or tools, we designed our own deep learning-based classification framework for traditional Chinese characters. It should be noted that the network training requires a very large dataset with varying forms of characters. Here, we employed “synthetic” characters with automatic labeling using several font families and a traditional Chinese handwriting dataset [27] to form a large dataset containing 1,202,564 images for network training and 300,641 images for validation. The resolution of each character is 64×64 pixels. After removing certain rare characters, there are 4155 Chinese characters for classification, which are commonly used in daily life.

**Figure 12:**
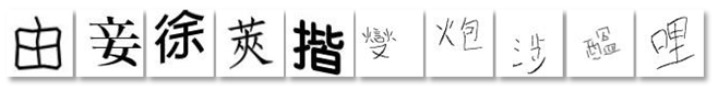
Some samples from our Chinese character dataset after data augmentation.

Since the number of types or classes of Chinese characters is much larger than that of English letters, appropriate data augmentation in training is critical. We employed a variety of transforms on the handwritten and printed characters, including random rotations, shearing, translation and perspective transformations. Figure 12 shows some examples after data augmentation, which significantly increases the diversity of characters.

The semantic and spatial information of the extracted feature map will affect the performance of classification in the deep neural network. Properly deepening and widening the feature network can effectively increase the model accuracy. After a thorough evaluation, we chose ResNeSt-50 [28] as our network architecture as it adopts a split-attention block structure, which does not require additional calculations when compared to the existing ResNet variants. It also incorporates the channel-wise attention strategy as well as multipath network layouts. ResNeSt also performs exceptionally well in image classification tasks.

## 5. Experimental Results

### 5.1. Experimental Results of Fingertip Detection

**Figure 13:**
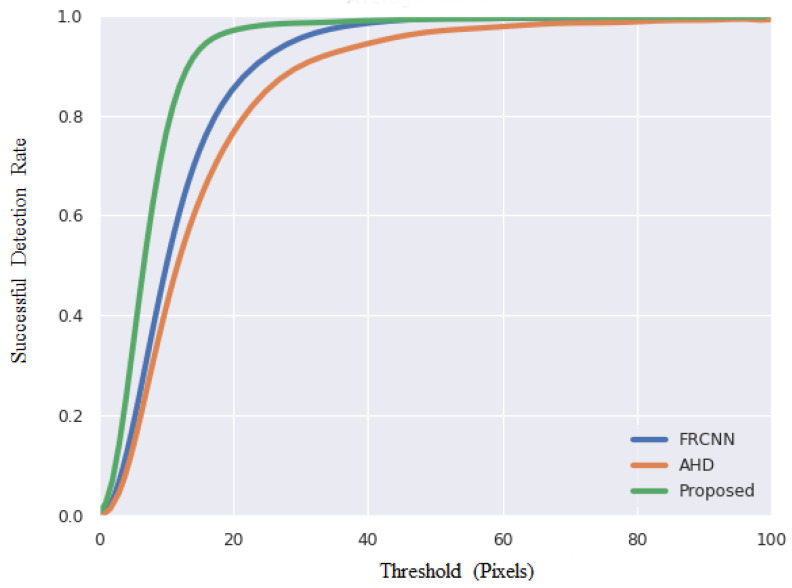
Comparison of fingertip detection under varying threshold values.

In order to compare the proposed scheme with existing methods, we used the same test dataset as in [14,15], which is known as the EgoFinger dataset, containing 12,974 images. Average Pixel Error (APE) was used as the evaluation metric, which indicates the pixel distance between the detected location and ground truth. Table 2 shows that the proposed scheme has a much smaller APE, demonstrating that it can accurately pinpoint the fingertip. Figure 13 shows the successful detection rates with different APE threshold values. A successful detection means that the distance between the detected fingertip from the ground-truth location is smaller than the threshold measured in pixels. In the proposed scheme, the threshold can be set smaller than 20 pixels and the successful detection rate can be higher than 90%.

**Figure 14:**
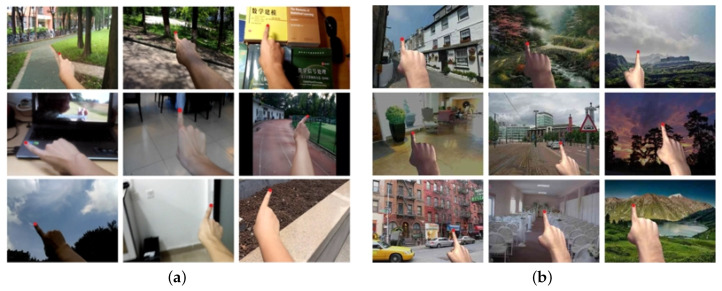
Examples of the proposed fingertip detection on: (**a**) the EgoFinger dataset; and (**b**) our synthetic dataset.

We also compared the results with those of four other algorithms: FRCNN [16], KCF [29], TLD [30], and MIL [31]. We set a threshold of 15 pixels to determine whether the detection is successful or not. Since the test dataset was not provided in these studies, we still used the EgoFinger test dataset in the experiments. The results are listed in Table 3. The precision and speed of the proposed scheme outperform those of the other four methods due to the customized design of the backbone network. Figure 14a,b shows some results on the EgoFinger dataset and our synthetic dataset, respectively. As we can see, the proposed scheme can correctly locate the fingertips in varying scenes.

### 5.2. Experimental Results of Chinese Character Recognition

To evaluate the accuracy of Chinese character recognition, we asked three different persons to use smart glasses to conduct a total of 400 air-writing experiments. We compared the results using two different training datasets, the synthetic dataset and the hybrid dataset. The synthetic dataset contains printed characters extracted from several font files. The hybrid dataset is a mixture of the synthetic dataset and the handwritten images from the Traditional Chinese Handwriting Dataset [27]. Table 4 shows that training with both datasets achieves satisfactory accuracy in Top 1, Top 5, and Top 10 cases. The mixture of synthetic data and real handwritten data does help to improve the performance. In our opinions, such mixtures introduce a variety of data, which increases the robustness of the proposed scheme.

Since there is currently no related work for Traditional Chinese character recognition, we used the well-known Google Cloud Vision API as a comparison with our results. The Google Cloud Vision API can automatically detect objects in images and supports detecting texts of over fifty languages, including Traditional Chinese characters. As indicated in Table 5, the current implementation of the proposed scheme outperforms Google Vision API significantly and has the Top 1 accuracy of up to 92%.

### 5.3. Air-Writing Examples

**Figure 15:**
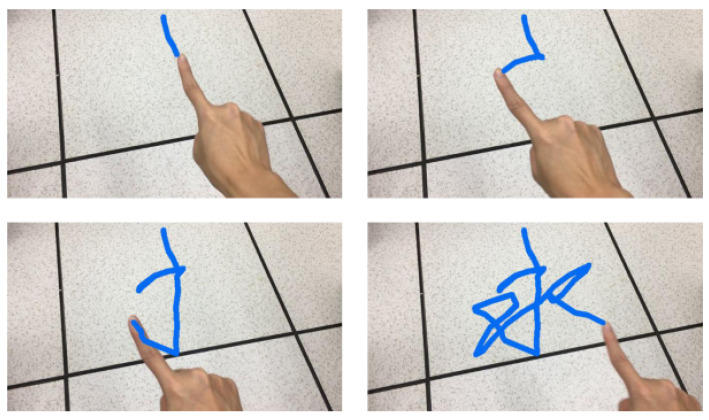
Testing characters for air-writing: We connect the fingertip coordinates to form the fingertip trajectories.

**Figure 16:**
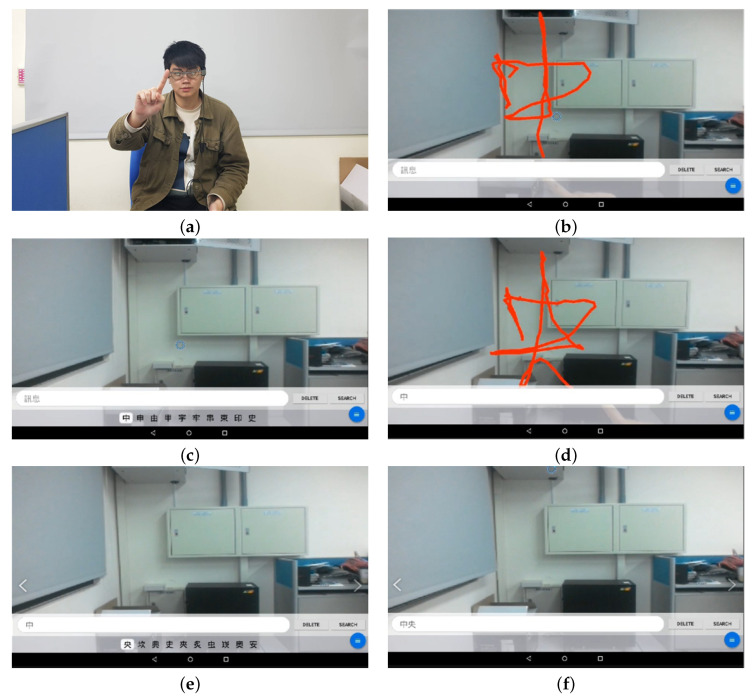
Example of air-writing for smart glasses: (**a**) a user wearing smart glasses writes characters in the air; (**b**) the writing trajectories of the first character; (**c**) the candidates are shown at the bottom of the the smart glasses screen; (**d**) the selected character is shown in the input text field and the user goes on writing the second character; (**e**) the candidates of the second character; and (**f**) the two characters are shown in the input text field.

Figure 15 shows the progress of fingertip tracking to form the writing trajectories of a character. The proposed air-writing scheme stores the fingertip coordinates and then connects the points to form the fingertip trajectories. Since the detection of fingertips is fast, the writing trajectories are smooth enough to form a recognizable character. Figure 16 further shows the operations of air-writing for smart glasses in a step-by-step manner. A user in Figure 16a wears smart glasses and writes characters (the word “Central” composed of two Chinese characters is used as an example) in the air as the input. Figure 16b shows the writing trajectories of the first character. It should be noted that the red trajectories are just superimposed in the figure for better illustration. The user does not see these red trajectories as they do not help the writing process. Figure 16c shows that the smart glasses display the first ten candidates, which are determined by the remote server, at the bottom of the screen for the user to select the correct one. After extensive tests, the first candidate is usually the one that the user writes. If not, the user may click the button to move the cursor to select other characters. The selected character is added to the input text field in Figure 16d. The user goes on to write the second character, and again the candidates are listed in Figure 16e. The word “Central” is then displayed in the text field, as shown in Figure 16f.

## 6. Conclusions and Future Work

A practical air-writing scheme for smart glasses is presented in this paper. A region-based convolutional neural network model is developed for real-time fingertip localization. MobileNetV2 is employed as the backbone network, which is further simplified by reducing the number of bottleneck layers to avoid redundant features. We use Unity3D to establish a synthetic dataset, avoiding manual labeling errors and providing a large benchmark dataset with high quality. The proposed scheme can detect and localize the fingertip in 640×480 RGB images, with 38.8 fps using GPU, and 8.31 average pixel errors. The writing trajectories can be effectively formed and the written character can be recognized by another classification network based on ResNeSt. The major contributions of the paper are: (1) the feasibility of using synthetic datasets only to achieve very good results of finger tracking in ego-centric-view images; and (2) adopting state-of-the-art deep learning networks to design customized finger detection and character recognition schemes for developing a new smart glasses human–computer interface for air-writing. Future work will be designing lightweight detection and recognition networks on mobile devices and increasing the adaptation of synthetic datasets to deal with other kinds of contents, such as third-person-perspective-view images, to further broaden the application scope. Besides, inviting users from different ethnic groups to test the scheme will be necessary so that the generality can be ensured.

## Figures and Tables

**Table 1:** Design of the backbone network design.

Input	Operator	t	c	n	s
640 × 640 × 3	Conv2D	-	32	1	2
320 × 320 × 32	Bottleneck	1	16	1	1
320 × 320 × 16	Bottleneck	6	24	1	2
160 × 160 × 24	Bottleneck	6	32	2	2
80 × 80 × 32	Bottleneck	6	64	3	2
40 × 40 × 64	Bottleneck	6	96	2	2

**Table 2:** Comparison on the EgoFinger dataset.

Method	Average Pixel Error
AHD [14]	15.72
FRCNN [15]	12.22
Proposed	8.31

**Table 3:** The results of precision and speed.

	Precision (15 px)	Speed (fps)
FRCNN [16]	73.1	18.5
KCF [29]	55.4	26.4
TLD [30]	66.7	10.6
MIL [31]	42.4	12.1
Proposed	93.3	38.8 (640p)

**Table 4:** Comparing the accuracy of character recognition using different training datasets.

	Synthetic Dataset	Hybrid Dataset
Top 1	84.25%	92%
Top 5	95%	97.5%
Top 10	97.75%	98.5%

**Table 5:** The results of precision and speed.

	Accuracy (Top1)
Google Vision API	33.5%
ResNeSt50 (ours)	92%

## Data Availability

Does not apply.
